# Effectiveness and Safety of Xen Gel Stent in Glaucoma Surgery: A Systematic Review of the Literature

**DOI:** 10.3390/jcm12165339

**Published:** 2023-08-16

**Authors:** Carlo Enrico Traverso, Roberto G. Carassa, Antonio Maria Fea, Michele Figus, Carlo Astarita, Benedetta Piergentili, Vanessa Vera, Stefano Gandolfi

**Affiliations:** 1 Eye Clinic, IRCCS San Martino Polyclinic Hospital, 16132 Genoa, Italy; mc8620@mclink.it; 2 Department of Neurosciences, Rehabilitation, Ophthalmology, Genetics, Maternal and Child Health (DiNOGMI), University of Genoa, 16126 Genoa, Italy; 3 Centro Italiano Glaucoma, 20124 Milan, Italy; carassa@glaucoma.it; 4 Department of Surgical Sciences, University of Turin, 10122 Turin, Italy; antoniomfea@gmail.com; 5 Department of Surgical, Medical and Molecular Pathology and Critical Care Medicine, University of Pisa, 56126 Pisa, Italy; 6 AbbVie S.r.l., 04011 Campoverde, LT, Italy; carlo.astarita@abbvie.com (C.A.); benedetta.piergentili@hotmail.it (B.P.); 7 AbbVie Inc., North Chicago, IL 60064, USA; vanessa.vera@abbvie.com; 8 Department of Medicine and Surgery, University of Parma, 43121 Parma, Italy; s.gandolfi@rsadvnet.it

**Keywords:** glaucoma, XEN, microinvasive filtering-surgery, trabeculectomy

## Abstract

Although topical medical therapy and selective-laser-trabeculoplasty represent the treatments of choice to reduce intraocular pressure, many patients do not achieve adequate glaucoma control; therefore, they require further options and eventually surgery. Trabeculectomy is still considered the gold standard, but the surgical management of glaucoma has undergone continuous advances in recent years, XEN-gel-stent has been introduced as a safer and less traumatic means of lowering intraocular pressure (IOP) in patients with open-angle glaucoma (OAG). This study aimed to review the effectiveness and safety of clinical data on XEN-stent in OAG patients with a Synthesis-Without-Meta-analysis (SWiM) methodology. A total of 339 studies were identified following a literature search adhering to PRISMA guidelines and, after evaluation, 96 studies are discussed. XEN63 and XEN45 device data were collected both short and long term. In addition, this document has evaluated different aspects related to the XEN implant, including: its role compared to trabeculectomy; the impact of mitomycin-C dose on clinical outcomes; postoperative management of the device; and the identification of potential factors that might predict its clinical outcomes. Finally, current challenges and future perspectives of XEN stent, such as its use in fragile or high myopia patients, were discussed.

## 1. Introduction

The term open-angle glaucoma covers a wide range of chronic and progressive optic neuropathies which have the loss of retinal ganglion cells and their axons in common, as well as the subsequent loss of the visual field [1].

Lowering intraocular pressure (IOP) is currently considered as the main known modifiable risk factor [2]. Topical hypotensive medication and selective laser trabeculoplasty are currently considered as the first treatment approaches in most patients [3]. However, some patients do not achieve adequate glaucoma control; therefore, they require further therapies and eventually surgery [3,4,5], such as trabeculectomy [6], which unfortunately may lead to potential vision-threatening complications [7].

Glaucoma surgery has experienced important advances over the last several years. 

One of the most important advances in glaucoma surgery in recent years was the development of the minimally or microinvasive glaucoma surgery (MIGS) devices [8]. They have been developed as safer and less traumatic means of lowering IOP in patients with glaucoma [8,9].

The definition of the term MIGS has been evolving since its introduction [9,10], and the generally accepted definition of MIGS has been changing over the years [11].

Among the different MIGS devices, XEN gel stents obtained the CE mark in December 2015 and were approved by the Food and Drug Administration (FDA) in November 2016 [12].

According to the classical definition, XEN is not defined as a MIGS because it is a bleb-forming device [9]; therefore, minimally invasive or micro-incisional filtration surgery have been suggested as more appropriate terms.

The aim of the current paper is to review the effectiveness and safety clinical data of XEN stent in open-angle glaucoma (OAG) patients.

### XEN Gel Stent

The XEN gel stent (AbbVie Inc., Chicago, IL, USA) was originally developed as an ab interno procedure that reduces IOP by draining aqueous fluid from the anterior chamber into the subconjunctival space [13,14,15]. Unlike other MIGS devices that target Schlemm’s canal and the supraciliary space to lower IOP, XEN gel stent was the first micro-incisional filtration surgical procedure to drain aqueous to subconjunctival space [14,15].

The stent is a hydrophilic tube that is 6 mm long, and it is composed of porcine gelatine crosslinked with glutaraldehyde to prevent degradation when implanted [13,16]. 

The XEN device is based on the Hagen–Poiseuille law of laminar flow, where the length and the inner diameter of the tube determine the flow resistance and, consequently, the flow rate. Three different devices with different inner diameters, namely 45, 63, and 140 μm, were investigated [13].

The 140 μm XEN device has not been commercialized to date and the evidence is limited to a single paper [17]. The evidence evaluating the clinical outcomes of the XEN63 device is very limited [18,19,20,21,22,23] and most studies were performed with an earlier version of the device injector that was never marketed [18,19,20,21]. The new XEN63 device uses the same needle injector as the XEN45 for preventing early sideflow and hypotony [22].

Although the XEN45 device was originally designed for ab interno implantation [13,14,15], surgeons have been gaining experience with the device, and different changes aiming to provide better clinical outcomes have been introduced in the implantation technique [24,25,26].

Systematic reviews are currently considered as an essential source of evidence when making decisions in the clinical management of patients [27]. However, there are some limitations in relation to the method used (Preferred Reporting Items for Systematic Reviews and Meta-Analyses [PRISMA]; http://www.prisma-statement.org/, accessed on 1 July 2023) [28,29]. Pooling data is not possible in all cases due to high levels of heterogeneity or lack of data [12,29]. In addition, there is a growing clinical demand to respond to complex questions or situations that incorporate various data sources due to their characteristics [30,31]. These facts have opened the door to a growing number of narrative syntheses of quantitative data. However, a major concern about such papers is lack of transparency and the consequent introduction of bias [32,33].

Synthesis Without Meta-analysis (SWiM) has emerged as a systematic review to address questions that meta-analysis may not be able to provide an adequate answer for [34].

This SWiM aims to examine the currently available scientific evidence to answer different clinical questions about the XEN device.

## 2. Materials and Methods

### 2.1. Information Sources and Search Strategy

This SWiM was carried out according to the guidelines of the of the PRISMA statement [35], although the current systematic review was not registered.

A group of Italian glaucoma specialists convened to review the currently available evidence about the efficacy and safety of XEN devices in patients with glaucoma. Searches of MEDLINE, the Cochrane Database, EMBASE, and Google Scholar were conducted using the search terms “Glaucoma”, “Open-angle glaucoma”, “XEN”, “MIGS”, “Combined surgery”, and “Gel implant”. References cited in selected articles were also reviewed to identify additional relevant reports. 

In addition, abstracts from the American Glaucoma Society, American Academy of Ophthalmology, European Glaucoma Society, and Association of Research in Vision and Ophthalmology were manually searched for relevant publications.

### 2.2. Study Selection, Data Extraction, and Synthesis Method

The authors independently generated the queries for the literature search and selected the articles fulfilling the criteria established for each subject and solved any disagreement through discussion and consensus. 

Limits were set for articles written in English, French, Spanish, Portuguese, and Italian with human subjects. The studies were published between August 2014 and April 2023.

Additionally, the following exclusion criteria were applied: (1) the study did not examine clinical outcomes or response to treatment of XEN device (any device); (2) the study was on subjects other than human adults; (3) the publication was a review article, an editorial, or an opinion piece.

To synthesize the results, we applied the SWiM guidelines [34].

## 3. Results

### 3.1. Results of the PRISMA Procedure

**Figure 1:**
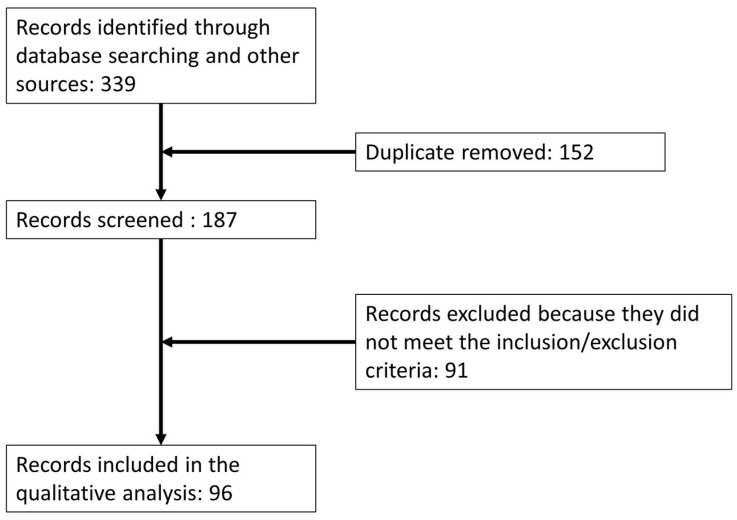
PRISMA 2009 flow diagram.

The steps of the literature search are summarized in the PRISMA 2009 flow diagram (Figure 1). A total of 339 records were identified after the initial search. After the removal of duplicates, 187 scientific papers remained. After careful review, 96 papers met all the requirements of the inclusion/exclusion criteria and were included for qualitative synthesis (Figure 1).

### 3.2. XEN63 

The currently available scientific evidence evaluating the clinical outcomes of XEN63 is very limited [18,19,20,21,22,23] (See Table 1), and most of the evidence was generated from a former device that was never commercialized; therefore, we will not go into detail analysing the results of those articles [18,19,20,21]. 

To date, there are two papers evaluating the efficacy and safety of the XEN63 device. Fea et al. [22] evaluated the new XEN63 device and found a mean (95% confidence interval, 95% CI) IOP lowering effect of −14.8 (−20.1 to −9.5) mmHg, *p* < 0.0001 at month-3, reporting that the mean IOP achieved with XEN63 was consistently lower than that obtained with XEN45. This may be an important point because the larger calibre of the Xen 63 device could by clinically meaningful for patients with high preoperative IOP. 

Additionally, Fea et al. [23] evaluated the effectiveness and safety of the new XEN63 device over a follow-up period of 18 months in patients with glaucoma in a real clinical setting. They reported significant IOP lowering and a reduction in the number of ocular hypotensive drugs during a follow-up period of 18 months. Moreover, the incidence rate of adverse events was relatively low and most of them were mild in severity [23].

Table 1 summarizes the main outcomes of XEN63 according to the current evidence.

### 3.3. XEN45

#### 3.3.1. Effect on Intraocular Pressure Lowering and Reduction in the Number of IOP-Lowering Medications

Multiple studies have evaluated the IOP lowering effect of the XEN device, either alone or in combination with cataract surgery, in patients with glaucoma [36,37,38,39,40,41,42,43,44,45,46,47,48,49,50,51,52,53,54,55,56,57,58,59,60,61,62,63,64,65,66,67,68,69,70,71,72,73,74,75,76,77,78,79,80,81,82,83,84,85,86,87,88,89,90,91,92,93,94,95,96,97,98,99,100,101]. Although most of the studies were performed in patients with primary open-angle glaucoma (POAG), other studies were performed in patients with pseudoexfoliative glaucoma (PXG) [40,42,54,72,98] or glaucoma secondary to uveitis [47].

The results of a systematic review and meta-analysis published recently, which used a pooled analysis with a random effects model, have shown a mean (95% CI) IOP lowering from baseline of −7.8 (−7.4 to −8.2) mmHg and −8.4 (−6.9 to −9.8) mmHg in the eyes of patients who underwent XEN-solo and XEN + Phaco, respectively. All patients were treated and followed up as routine clinical practice between May 2013 and February 2020. The mean sample size was 79 ± 67 and the average follow-up time was 17.0 ± 8.1 months [12].

Similarly, Yang et al. [102] did not find significant differences in IOP lowering between XEN-solo and XEN + Phaco (standardized mean difference: −0.01, 95% CI −0.09 to 0.08, *p* value 0.894). Moreover, Panarelli et al. [103] reported that, on average, successful gel stent surgery achieved a postoperative IOP of approximately 14.0 mm Hg and reduction to fewer than 1 ocular hypotensive medication.

Regarding the number of ocular hypotensive medications (See Table 2, Table 3 and Table 4), Chen et al. [12] reported a significant reduction in the number of ocular hypotensive drugs in both XEN-solo procedures (Mean: −1.97 drugs; 95% CI: −2.19 to −1.75 drugs, *p* < 0.001) and in the XEN + Phaco ones (Mean: −1.86 drugs; 95% CI: −2.11 to −1.60 drugs, *p* < 0.001).

Similar results have been published by Yan et al. [102], who found a statistically significant reduction in ocular hypotensive medications (standardized mean difference: 2.11, 95% CI 1.84 to 2.38, *p* value <  0.001). Furthermore, after adjusting for different covariates, the reduction in the number of ocular hypotensive drugs was significantly lower in the XEN + Phaco group than in the XEN-solo group (Risk ratio: 1.45, 95% CI 1.06 to 1.99, *p* value 0.019) [102].

##### IOP Lowering at 12 Months 

Thirty-four studies have evaluated the IOP lowering effect of the XEN device over 12 months of follow-up (see Table 2). On average, the results of the different studies pointed in the same general direction, mostly indicating that XEN45 provided IOPs below 15 mmHg after one year of follow-up (Table 2), which is consistent with the most recent meta-analysis published [12,101,102].

##### IOP Lowering at 24 Months

Twelve studies have evaluated the IOP lowering effect of XEN45 after 24 months of follow-up (See Table 3). The results of the different studies included in this SWiM paper have shown that, after 2 years of follow-up, XEN45 provided a good IOP lowering, with final IOPs ≤15 mmHg and an IOP lowering that ranged between −5 and −10 mmHg (Table 3).

##### IOP Lowering at 36-Months

Six papers have evaluated the longer-term efficacy of XEN45 in terms of IOP lowering and reducing the number of ocular hypotensive medications, (See Table 4). With the exception of one paper (n = 23 eyes), which reported an IOP of 19.6 ± 2.1 mmHg at 36 months [78], currently available evidence shows good hypotensive effects for XEN45, with IOPs at 36–48 months in the range of 13–14 mmHg, as well as a significant reduction in the number of ocular hypotensive medications [45,61,81,87,88,94,101]. In addition, compared to preoperative values, the reduction in IOP was greater than 30% (Table 4).

Current evidence has demonstrated the efficacy and safety of the XEN45 device in the short, medium, and long term.

#### 3.3.2. Can XEN45 Be Safely and Effectively Implanted in Myopic Patients?

Evidence surrounding the use of XEN45 in myopic OAG patients is limited [52,64,104,105,106]. In fact, there are currently only two studies that specifically evaluated the efficacy and safety of XEN45 in patients with high myopia.

Laborda-Guirao et al. did not find significant differences between OAG eyes with or without high myopia in intraocular pressure (IOP) lowering, success rate, reduction in the number of ocular hypotensive medications, or postoperative complications, which clearly suggested that XEN45 may be safely and effectively used in glaucomatous eyes with high myopia [52].

Chao et al. [64], have retrospectively evaluated the effectiveness and safety of the XEN45 in East Asian patients with primary open angle glaucoma (POAG). Although this study did not specifically evaluate the efficacy and safety of XEN45 in patients with high myopia, the mean spherical equivalent was −5.13 ± 4.44 diopters, with a range of −13.63 to −2.88 diopters and mean axial length of 26.67 ± 1.65 mm (up to 29.34 mm). According to the results of this study, axial length was not significantly associated with success, either complete (odds ratio: 0.082, *p* = 0.559) or qualified (odds ratio: 0.659; *p* = 0.186); need of subsequent intervention (odds ratio: 0.959, *p* = 0.803); or need of additional surgery (odds ratio: 1.382, *p* = 0.382). Additionally, no major complications were observed [64]. These results suggested that axial length did not have any influence on XEN45 outcomes.

Sacchi et al. [105], in a retrospective study (which included seven eyes followed for 2 years) evaluated the effectiveness and safety of Xen 45 in patients with medically uncontrolled glaucoma and concomitant high myopia (>6 Diopters). Preoperative mean IOP was significantly lowered from 22.1± 4.9 mmHg to 14.8 ± 4.0 mmHg at month-24, *p* < 0.0001. Regarding safety, two eyes had hypotony (without maculopathy) and one eye had choroidal detachment [106]. According to the results of this study, the XEN implant had a better safety profile than trabeculectomy, with a similar hypotensive profile [105].

Additionally, Fea et al. assessed the effectiveness and safety of XEN in 31 glaucomatous eyes with a refractive error higher than −6 D and an axial length ≥26 mm [106]. Mean preoperative IOP (95% CI) was significantly lowered from 23.5 (20.5–26.4) mm Hg to 13.0 (12.2–13.8) mm Hg, *p* < 0.0001. Regarding safety, hypotony (an IOP <6 mm Hg) was reported in eight eyes (28.6%) during the first postoperative day and remained for a week [106].

Conversely, one publication described the clinical outcomes of XEN45 in a high myopic eye. The patient required a XEN removal to control IOP, which resulted in loss of the remaining visual field in the eye [104].

Currently available scientific evidence suggests that XEN45 may be effectively and safely implanted in myopic eyes. However, there is a need for a prospective study specifically evaluating the clinical outcomes of XEN45 in eyes with OAG and high myopia.

#### 3.3.3. XEN-Solo Versus XEN + Phaco: Is There a Difference in Terms of IOP Lowering?

This question has brought an increasing interest among glaucoma specialists.

Fifteen papers have compared the efficacy of XEN45 solo or in combination with cataract surgery (phacoemulsification). After reviewing the literature, the most plausible conclusion is that there is no agreement regarding the superiority of the solo procedure over the combined procedure with cataract surgery (See Table 5).

According to the results of Chen et al. [12], both XEN alone (mean difference: −7.8 mmHg; 95% CI: −8.21 to −7.38 mmHg, *p* < 0.001) and XEN + Phaco (mean difference: −8.35 mmHg; 95% CI: −9.82 to −6.88 mmHg, *p* < 0.001) significantly lowered the IOP. 

Similarly, Yang et al. [103], in a systematic review and metanalysis, did not find significant differences between XEN and phaco-XEN surgery in terms of IOP after surgery (standardized mean difference: −0.01, 95% CI −0.09 to 0.08, *p* value 0.894). Nevertheless, the reduction in the number of ocular hypotensive medications was greater in the XEN solo group (*p* = 0.019) [102].

However, another systematic review and meta-analysis published by Wang et al. [107] has shown different results. They found better results for XEN alone compared to XEN + Phaco procedures in IOP lowering, but not in reducing ocular hypotensive medications [107].

In light of the available scientific evidence and based on our own experience, the XEN45 device, either alone or in combination with cataract surgery (phacoemulsification), significantly lowers IOP and reduces the number of ocular hypotensive medications. Similar to trabeculectomy, there are no data to support the superiority of the combined surgery over the solo surgery, and vice versa.

#### 3.3.4. XEN Versus Trabeculectomy: Is There a Difference in Terms of IOP Lowering?

Due mainly to its well-stablished IOP lowering effect, trabeculectomy is currently considered as the gold standard in glaucoma surgery [5]. However, it may lead to potential vision-threatening complications [6].

The Gold-Standard Pathway Study (GPS) [108] was the first multicenter, prospective, and randomized study comparing the effectiveness and safety of XEN45 versus trabeculectomy in glaucoma that was poorly controlled with topical IOP-lowering therapy. The results of this study showed that the mean IOP change from baseline was significantly greater at month 12 in the trabeculectomy group (0.024), although both treatments significantly lowered IOP from preoperative values (*p* < 0.001). XEN and trabeculectomy significantly reduced the preoperative mean number of ocular hypotensive medications, without significant differences between them (*p* = 0.068).

In addition, XEN was noninferior to trabeculectomy, regarding the prespecified primary endpoint (62.1% and 68.2% achieved the primary endpoint, respectively; *p* = 0.487), namely the percentage of patients achieving ≥20% IOP reduction from baseline at Month 12 without medication increase, clinical hypotony, vision loss to counting fingers, or secondary surgical intervention. Finally, XEN resulted in less need for in-office postoperative interventions (*p* = 0.024 after excluding laser suture lysis), faster visual recovery (*p* < 0.05), and greater 6-month improvements in visual function problems (*p* ≤ 0.022) [108]. 

Similarly, Marcos-Parra et al. [48] compared the IOP lowering effect and the reduction in ocular hypotensive medications between the XEN device and trabeculectomy in OAG patients. They reported a significantly greater IOP lowering in the trabeculectomy group than in the XEN group (*p* = 0.001), with a similar reduction in the ocular hypotensive drugs [48]. However, this difference was mainly due to the combined surgery (glaucoma + cataract). When comparing the IOP lowering effect between XEN + Phaco and Trabeculectomy + Phaco, IOP lowering from preoperative values was found to be significantly greater in the Trabeculectomy + Phaco group at day 1, week 1, and months 1 and 3. While comparing XEN alone versus trabeculectomy alone, the only significant differences were observed in month 6. Moreover, in terms of success rates, there were no differences in the proportion of eyes who achieved a final IOP ≥6 and ≤16 mmHg (*p* = 0.1317), although it was slightly lower in the XEN (66.2%) than in the trabeculectomy group (78.6%) [48].

Additionally, Schlenker et al. [109] observed similar rates of complete and qualified success for both interventions.

Wang et al. [102], in a systematic review and meta-analysis, reported no significant differences between XEN gel implant and trabeculectomy on lowering IOP, although the analysis showed a high heterogeneity (I2:60%). However, after a sensitive analysis that excluded the Schenkler et al. study [109] and reduced the heterogeneity (I2:0%), the results showed that trabeculectomy was more effective in lowering IOP, without significant differences in terms of the reduction in ocular hypotensive medications [102].

Table 6 shows a comparison of the clinical outcomes of XEN45 and Trabeculectomy.

Compared to trabeculectomy, XEN45 has been associated with several advantages, including less conjunctival manipulation, less postoperative inflammation, and lower incidence of postoperative adverse events. Moreover, the rates of postoperative interventions seem to be lower with XEN45 than with trabeculectomy. Therefore, XEN45 could be considered as the first-choice surgery in certain types of patients.

#### 3.3.5. XEN Implant: What Dose of Mitomycin-c Is the Most Effective?

Mitomycin-c (MMC) and 5-fluorouracil (5-FU) have been commonly used in traditional glaucoma filtration surgery, with good evidence suggesting a significant increase in surgery success; however, there is also an increase in the risk of complications [112].

Although MMC was not initially used in all studies [17,19,21], the use of intraoperative MMC has become a common practice of the XEN implant surgical technique [36,37,38,39,40,41,42,43,44,45,46,47,48,49,50,51,52,53,54,55,56,57,58,59,60,61,62,63,64,65,66,67,68,69,70,71,72,73,74,75,76,77,78,79,80,81,82,83,84,85,86,87,88,89,90,91,92,93,94,95,96,97,98,99,100,101].

Regarding XEN45, MMC concentration may influence clinical outcomes. It has been suggested that the success rate may be related to the MMC dose, although other studies have shown a lack of correlation between MMC dosage and the surgical outcomes [113]. In general terms, the use of MMC seemed to increase the therapeutic success rate after XEN45 gel stent implantation, although the ideal dose has not been stablished yet.

Selection of MMC concentration is based on a patient-tailored approach. This may entail an important bias because surgeons will use higher concentrations of MMC in more complicated cases in which they anticipate that the possibility of surgical failure is greater.

A study comparing the efficacy and safety of two MMC doses (0.01% versus 0.02%) in eyes who underwent a XEN45 device implant, either alone or in combination with phacoemulsification, was recently published [95]. The results of this study found that IOP lowering, number of hypotensive medication reduction, or incidence of adverse events were not related to MMC concentration [95]. The lack of significant differences, in terms of IOP lowering or reducing hypotensive medications, between MMC 0.01% and MMC 0.02% raises the possibility of using lower doses of MMC. However, it should be mentioned that MMC 0.01% was not associated with lower incidence rates of adverse events [95].

So far, there is no evidence to recommend the use of a specific MMC concentration. It seems that MMC dose does not significantly impact either the IOP lowering or the reduction in the number of ocular hypotensive medications, so it would seem prudent to recommend “the lowest dose of MMC that, in the surgeon’s opinion, may be effective in that patient”. However, new studies will be necessary to clarify this issue.

#### 3.3.6. XEN 45 Device Implant: Postoperative Bleb Management 

Bleb fibrosis is a common complication that may occur after a XEN implant, with rates as high as 45% [114]. Needling is a minimally invasive procedure that is commonly used for restoring the functionality of failed filtering blebs [115]. 

The most frequently reported postoperative intervention in eyes who underwent a XEN device is needling of the conjunctival bleb, which ranges from 5% to 62% [12] (Table 2, Table 3 and Table 4 show the needling rates reported in the different studies). 

Needling is not currently considered as an additional procedure, but rather as part of the normal bleb management in XEN implant surgery (similarly to laser suture lysis in trabeculectomy). In most cases, needling is required within the first month postoperatively.

However, in most studies, needling has been used as a rescue strategy once bleb failure has occurred.

Primary needling, at the time of ab interno XEN implantation, has been recently proposed as a technique that may reduce the number of postoperative interventions [96,116].

In a retrospective study, Kerr et al. [116] reported that primary needling at the time of XEN insertion was associated with a significant reduction in the number of bleb interventions (*p* = 0.003) and, consequently, the subsequent postoperative visits (*p* = 0.043). Additionally, compared to preoperative values, this technique has provided a significant reduction in both IOP and ocular hypotensive medication [116].

More recently, Buenasmañanas-Maeso et al. [96] performed a retrospective study that assessed the efficacy and safety of primary needling in eyes who underwent a XEN45 implant, either alone or in combination with phacoemulsification. According to the results of this study, primary needling was associated with fewer postoperative interventions. Nevertheless, these eyes were not associated with greater IOP lowering or a greater reduction in ocular hypotensive therapy [96].

Regarding the use of primary needling, there is not enough evidence to recommend its use on a routine basis. Therefore, from a clinical point of view, its use was reserved for those cases in which the XEN implant was twisted, trapped, or did not appear free and mobile in the subconjunctival space. Additionally, it may eventually be used in eyes with previously failed glaucoma surgery or in eyes with pathologies that may be associated with an increased risk of conjunctival fibrosis.

#### 3.3.7. Can XEN45 Device Be Considered a Safe Procedure?

Although XEN has emerged as a safer and less traumatic approach for lowering IOP in patients with glaucoma, it is not free of complications. 

According to the currently available scientific evidence, transient hypotony (defined as IOP <6 mmHg) is the most commonly reported complication of XEN45, with an incidence rate of 9.59%. In the vast majority of patients, hypotony is successfully resolved without additional surgery interventions and the rate of chronic hypotony is very low [12].

The second most common adverse event is hyphema (5.53%). Most of patients have grade I hyphema (less than 1/3 of anterior chamber), which had resolved spontaneously by the first week after surgery [12]. The third most commonly reported adverse event is the incidence of transient IOP spikes, which have been reported in 2.11% of eyes (in most cases associated with hyphema) [12].

**Figure 2:**
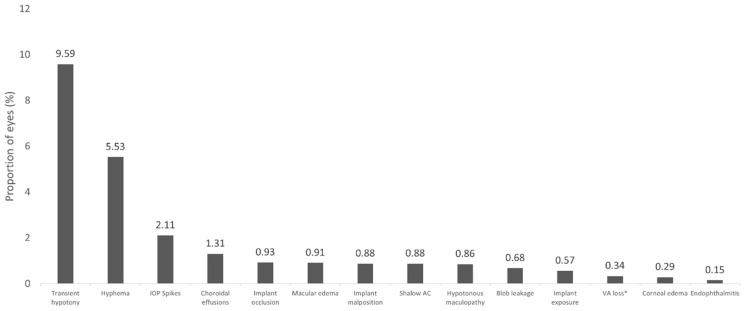
Incidence rate of adverse events reported after XEN implantation (*n* = 4410). Adapted from Chen et al. [12]. * ≥2 Snellen lines vison loss lasting >1 month. IOP: intraocular pressure; AC: anterior chamber; VA: visual acuity.

Figure 2 shows an overview of the incidence of different adverse events reported after XEN implantation. Regarding the number of corneal endothelial cells, trabeculectomy and glaucoma drainage device implantation can damage corneal endothelial cells [117,118,119,120]. With regards to the impact of XEN on the corneal endothelial cells’ loss, there were no significant differences between XEN + phacoemulsification and phacoemulsification alone [121,122,123]. Additionally, the corneal endothelial cell density reduction after XEN implantation as a solo procedure was low [122].

The results of a prospective, cross-sectional, and non-randomized clinical trial suggested that, in eyes who underwent a XEN standalone procedure, there were no significant changes in endothelial cell counts over the follow-up period (5 years). However, a statistically significant reduction in the central endothelial cell count was observed in the eyes who underwent a combined procedure (XEN + phacoemulsification) at different timepoints. This clearly suggests that the loss of corneal endothelial cells seems to be related with the cataract surgery, rather than with the XEN implant [123].

Both the experience of the panel and the scientific evidence have shown an acceptable safety profile of the XEN implant. The rate of serious complications (endophthalmitis, hypotonus maculopathy, corneal or macular edema) is low. Moreover, compared to trabeculectomy, the XEN implant has provided a better safety profile [93].

#### 3.3.8. XEN45 Device: Is It Possible to Predict Its Clinical Outcomes?

Different studies have evaluated potential predictive factors for failure [19,42,50,70,98] or for success [52,64,116].

Three studies [42,50,70] found that none of the evaluated factors were significantly associated with surgery failure, whereas two studies reported that the risk of failure is greater in men than in women [19,98]. However, Gabbay et al. [87] reported that women were more likely to fail. Gillmann et al. [98] observed that eyes with a primary OAG diagnosis and those requiring needling are more likely to fail.

Fea et al. [70] suggested that patients with lower IOP at day 1 and month 1 had a higher chance of success. The same was true if the difference between month 1 and week 1 IOP was lower than 6 mmHg.

Regarding surgery success, each mmHg increase in preoperative IOP was positively associated with surgery success (Odds ratio: 1.33; 95% CI: 1.13 to 1.55, *p* = 0.0004) [52].

Chao et al. [64] reported that surgical success was more likely for eyes with a better preoperative visual field mean deviation and lower IOP at day 1, week 2, and month 1. 

Conversely, Ibáñez-Muñoz et al. [116] found that none of the analysed factors were statistically associated with success.

The question of whether patient ethnicity impacts the XEN clinical outcomes has not been fully elucidated. Success rates appear to be lower in Black and Afro-Latino patients than in Caucasian populations [12,51,56]. Similarly, it has been observed that the ethnic Chinese group presents a reoperation rate as high as 45.9% [64]. However, other studies have suggested that there is no relationship between the ethnic origin of the patients and the clinical outcomes of XEN [39,62].

## 4. Discussion

The objective of this article was to address, in the most practical way possible, different aspects related to the XEN device that may generate doubts, mainly for specialists who are beginning to use this technique.

Based on currently available evidence, the XEN45 device, either alone or in combination with cataract surgery, significantly lowers IOP and reduces the number of ocular hypotensive medications in patients with glaucoma [12,36,37,38,39,40,41,42,43,44,45,46,47,48,49,50,51,52,53,54,55,56,57,58,59,60,61,62,63,64,65,66,67,68,69,70,71,72,73,74,75,76,77,78,79,80,81,82,83,84,85,86,87,88,89,90,91,92,93,94,95,96,97,98,99,100,101,108,110,111,116]. There are promising results with the new XEN63, but the evidence is very limited [22,23].

Compared to trabeculectomy [12,48,58,65,72,84,108,109,110,111], XEN seems not to be comparable in terms of IOP lowering, although XEN has similar efficacy in reducing the number of ocular hypotensive medications and provided a better safety profile.

Regarding its implantation technique, XEN has usually been delivered using an ab interno approach through a corneal incision [36,37,38,39,40,41,42,43,44,45,46,47,48,49,50,51,52,53,54,55,56,57,58,59,60,61,62,63,64,65,66,67,68,69,70,71,72,73,74,75,76,77,78,79,80,81,82,83,84,85,86,87,88,89,90,91,92,93,94,95,96,97,98,99,100,101]. However, as surgeons have been gaining experience with the device, different changes that aim to provide better clinical outcomes have been introduced in the implantation technique [24,25,26].

High myopia represents a challenging scenario in glaucoma patients who need surgical treatment. There are two studies that assessed the efficacy and safety of XEN in patients with high myopia. The results of both studies have pointed in the same direction, clearly indicating that the XEN device is an effective and safe option in patients with high myopia [105,106].

Trabeculectomy is still considered as the gold standard in glaucoma surgery [5]. It effectively lowers IOP and reduced the need of ocular hypotensive medications [6]. However, it requires a close postoperative follow-up to prevent potential complications that may lead to severe vision loss [7,124,125,126].

Nevertheless, XEN has been associated with lower conjunctival manipulation and incidence of postoperative complications and adverse effects [12,48,93,100].

For precisely this reason, this need for postoperative conjunctival manipulation has been one of the main points of discussion of XEN. The vast majority of the studies show a rate of needling which ranges from approximately 20 to 40% (See Table 2 and Table 3).

Primary needling (at the time of ab interno XEN implantation) has emerged as a valuable option to reduce the number of postoperative interventions [96,116].

Regarding the existence of predictive factors of the XEN clinical outcomes, current evidence does not show conclusive results.

Finally, regarding the impact of the XEN device on the corneal endothelium, current evidence suggests that XEN does not have a significant impact on corneal endothelial cell loss [121,122,123], unlike what occurs with trabeculectomy or other drainage devices [117,118,119,120].

## 5. Conclusions

According to published evidence, the XEN45 device lowers IOP by approximately 35% from preoperative values, obtaining a mean IOP value of ≤15 mmHg, as far as 4 years after surgery. In addition, XEN significantly reduced the need for ocular hypotensive medication, with a mean number of postoperative hypotensive medications ≤ 1 drug.

Based on the evidence and panel’s opinion, the XEN device may be considered as the first surgical option in patients who require a target IOP in the mid to low teens. Even though trabeculectomy seems to have a better IOP-lowering effect, the XEN device has been shown to have a better safety profile.

Although XEN device implantation is a relatively new procedure, a large number of studies have been published in recent years, pointing to its long-term potential in the treatment of glaucoma. 

Several issues related to XEN clinical outcomes remain to be clarified, such as the role of ethnicity, factors influencing the outcomes, needling rates and the role of primary needling, the impact of high myopia, and the use of XEN in different types of glaucoma, including narrow-angle and angle-closure glaucoma.

Data from randomized and multicentre clinical trials will help surgeons develop patient-tailored management strategies.

## Figures and Tables

**Table 1:** An overview of currently available scientific evidence with the XEN63 device.

Study	Follow-Up	Preop IOP, mmHg	Final IOP, mmHg	IOP Lowering	Mean Preoperative NOHM	Mean NOHM, Last Visit	Success Rates, (%) ^1^
Fea et al. [22]	3 months	27.0 ± 7.8 *	12.2 ± 3.4 *	−14.8 (−20.1 to −9.5) **	2.3 ± 0.9 *	0.1 ± 0.4 *	69.6
Fea et al. [23]	18 months	27.0 ± 7.8 *	14.1 ± 3.4 *	−12.9 (−16.9 to −8.9) **	2.3 ± 0.9 *	1.0 ± 1.4 *	77.8

* Mean (Standard deviation). ** Mean (95% confidence interval). ^1^ Complete success. Preop: preoperative; IOP: intraocular pressure; NOHM: number of ocular hypotensive medications.

**Table 2:** A comparison of the clinical outcomes of XEN45 after 12 months of follow-up.

Study	Type of Study	N	Preop IOP, mm Hg	M12 IOP, mm Hg	M12 IOP Lowering	Mean Preoperative NOHM	Mean NOHM, Last Visit	Needling Rates at Last Follow-Up Visit, n (%)
Ozal et al. [37] ^1^	Retrospective	15	36.1 ± 3.7 *	16.7 ± 3.6 *	−19.3 ± 5.0 *	3.6 ± 0.5 *	0.3 ± 0.9 *	Not reported
Galal et al. [38]	Prospective	13	16.0 ± 4.0 *	12.0 ± 3.0	23 ^⁑,†^	1.9 ± 1.0 *	0.13 ± 0.11 *	4 (30.7)
Grover et al. [39]	Prospective	65	25.1 ± 3.7 *	15.9 ± 5.2 *	−9.1 (−10.7 to 7.5) **	3.5 ± 1.0 *	1.7 ^†^	21 (32.3)
Hengerer et al. [40]	Retrospective	242	32.2 ± 9.1 *	14.2 ± 4.0	32.2 ^⁑,†^	3.1 ± 1.0 *	0.3 ± 0.7 *	67 (27.7) ***
De Gregorio et al. [41]	Prospective	33	22.5 ± 3.7 *	13.1 ± 2.4 *	−9.4 ± 3.1 *	2.5 ± 0.9 *	0.4 ± 0.8 *	Not reported
Mansouri et al. [42]	Prospective	149	20.0 ± 7.1 *	13.9 ± 4.3	−31 ^⁑,†^	1.9 ± 1.3 *	0.5 ± 0.8 *	55 (36.9)
Widder et al. [44]	Retrospective	261	24.3 ± 6.6 *	16.8 ± 7.6 *	−7.5 ± 7.1 *	2.6 ± 1.1 *	0.2 ± 0.7 *	80 (34)
Reitsamer et al. [45]	Prospective	202	21.4 ± 3.6 *	14.9 ± 4.5 *	−6.5 ± 5.3 *	2.7 ± 0.9 *	1.1 ± 1.2 *	83 (41.1)
Kalina et al. [47]	Prospective	47	22.3 ± 7.3 *	13.4 ± 3.6 *	−8.9 ± 5.8 *	3.0 ± 1.2 *	0.8 ± 1.3 *	14 (29.8)
Marcos-Parra et al. [48]	Retrospective	65	19.1 ± 5.4 *	N.A.	−6.7 (−12.9 to −0.5) **	2.5 ± 0.8 *	0.2 ± 0.6 *	13 (20.0)
Sng et al. [50]	Prospective	31	15.6 ± 2.7 *	12.1 ± 2.6 *	−3.5 ± 2.7 *	1.4 ± 0.6 *	0.1 ± 0.4 *	Not reported
Gabbay et al. [51]	Retrospective	151	22.1 ± 6.5 *	15.4 ± 5.9 *	−6.7 ± 6.2 *	2.77 ± 1.1 *	0.5 ± 1.0 *	57 (37.7)
Laborda-Guirao et al. [52]	Retrospective	80	21.0 ± 5.2 *	14.7 (13.9 to 15.4) **	−6.3 (−8.8 to −4.4) **	2.8 (2.7 to 3.0) **	1.1 (0.8 to 1.3) **	7 (8.8)
Heidinger et al. [53]	Retrospective	199	22.8 ± 6.9 *	17.1 ± 5.9 *	−5.7 ± 6.6 *	2.9 ± 1.0 *	1.8 ± 1.4 *	44 (22.1)
Ibáñez-Muñoz et al. [54]	Retrospective	21	22.3 (21.0−23.5) **	15.3 (14.3−16.3) **	−7.3 (−9.7 to −5.0) **	3.0 ± 1.0 *	1.2 ± 1.2 *	19 (26.0)
Karimi et al. [55]	Retrospective	259	19.3 ± 6.0 *	14.3 ± 4.4 *	−5.1 ± 5.6 *	2.6 ± 0.1 *	1.6 ± 0.5 *	106 (40.9) ^2^
Laroche et al. [56]	Retrospective	20	15.3 ± 6.2 *	12.9 ± 4.5 *	−2.4 ± 5.4 *	3.6 ± 0.7 *	1.8 ± 1.5	0 (0.0)
Smith et al. [57]	Retrospective	68	22.1 ± 6.4 *	14.8 ± 5.1 *	−7.3 ± 5.8 *	2.9 ± 0.8 *	1.1 ± 1.1 *	30 (44.1)
Mansouri et al. [59]	Prospective	109	20.0 ± 7.5 *	N.A.	N.A.	2.0 ± 1.3 *	0.6 ± 0.9 *	58 (45)
Post et al. [61]	Prospective	20	21.6 ± 2.3 *	17.7 ± 2.1 *	−3.9 ± 2.2 *	3.2 ± 0.8 *	1.6 ± 1.0 *	5 (25.0)
Chao et al. [64]	Retrospective	37	21.7 ± 7.7 *	15.0 ± 2.0 *	−6.7 ± 5.6 *	3.4 ± 0.9 *	1.3 ± 1.5 *	17 (45.9)
Schargus et al. [66]	Retrospective	153	23.9 ± 7.4 *	15.4 ± 5.1 *	−8.5 ± 6.4 *	2.6 ± 1.2 *	0.8 ± 1.3 *	64 (35.3)
Theilig et al. [67]	Retrospective	100	24.5 ± 6.7 *	16.6 ± 4.8 *	N.A.	3.0 ± 1.1 *	1.4 ± 1.5 *	42 (42.0)
Subaşı et al. [69]	Retrospective	30	20.4 ± 4.8 *	15.0 ± 1.9 *	−6.2 ± 0.9 *	3.1 ± 1.0 *	0.9 ± 1.1 *	13 (43.3)
Fea et al. [70]	Prospective	171	23.9 ± 7.6 *	15.5 ± 3.9 *	−7.4 ± 7.9	3.0 ± 1.0 *	0.5 ± 1.0 *	79 (46.2)
Rauchegger et al. [71]	Retrospective	79	23.4 ± 7.9 *	14.6 ± 3.6 *	31(20−42) ^⁑,⁋^	2.7 ± 1.1 *	1.0 ± 1.2 *	37 (62)
Eraslam et al. [81]	Retrospective	26	23.9 ± 6.8 *	16.4 ± 2.6 *	−7.5 ± 5.2 *	2.9 ± 0.7	0.9 ± 0.9	21 (36.2)
Lewczuk et al. [83] ^3^	Retrospective	86	25.0 ± 7.5 *	16.8 ± 5.1 *	−8.2 ± 6.4 *	Not reported	Not reported	30 (69.8)
Wanichwecharungruang and Ratprasatporn [84]	Retrospective	57	21.6 ± 4.0	15 ^†^	−30.6 ^⁑,†^	2.1 ± 1.4 *	0.5 ± 0.7 *	10 (17.5)
Reitsamer et al. [86]	Retrospective	212	20.7 ± 5.1	14.8 ^†^	−5.6 ^†^	2.5 ± 1.0 *	0.7 ± 1.0 *	78 (36.8)
Nicolau et al. [89]	Retrospective	186	18.1 ± 5.1 *	13.7 ± 5.6 *	−4.4 ± 5.3 *	2.5 ± 1.1 *	0.8 ± 1.0	25 (13%)
Monja-Alarcón et al. [95]	Retrospective	63	17.6 (0.7) ^⁂^	12.6 (0.3) ^⁂^	21.9 ± 27.6 ^⁑^	2.1 (1.9 to 2.3) ^⁋^	0.2 (0.04 to 0.3) ^⁋^	19 (30.2)
Buenasmañanas-Maeso et al. [96]	Retrospective	63	17.6 ± 5.3 *	12.6 (12.0 to 13.3) ^⁋^	−21.9 ± 27.4 ^⁑^	2.1 (1.9 to 2.3) ^⁋^	0.2 (0.04 to 0.3) ^⁋^	8 (12.7)
Almendral et al. [99]	Retrospective	63	17.6 ± 5.3 *	12.6 ± 2.6 *	−22.0 ± 27.5 ^⁑^	2.1 ± 0.7 *	0.2 ± 0.5 *	7 (11.1)

^†^ Data about standard deviation was not provided. * Mean (Standard deviation). ** Mean (95% confidence interval). ^⁑^ Percentage. ^⁂^ Mean (Standard error). ^⁋^ 95% Confidence interval. *** All the needling procedures were done between week 1 and month 3. ^1^ Data calculated from Table 2 of the paper. ^2^ Postoperative bleb needling or antimetabolite injection. ^3^ Data from naïve patients. Abbreviations: IOP: intraocular pressure; N: number of eyes; NOHM: number of ocular hypotensive medications.

**Table 3:** A comparison of the clinical outcomes of XEN45 after 24 months of follow-up.

Study	Type of Study	N	Preop IOP, mm Hg	M 24 IOP, mm Hg	M 24 IOP Lowering, mm Hg	Mean Preoperative NOHM	Mean NOHM, Last Visit	Needling Rates at Last Follow-Up Visit, n (%)
Reitsamer et al. [45]	Prospective	202	21.4 (3.6) *	15.2 ± 4.2 *	−6.2 ± 4.9 *	2.7 ± 0.9 *	1.1 ± 1.2 *	83 (41.1)
Gabbay et al. [51]	Retrospective	151	22.1 ± 6.5 *	14.5 ± 3.3 *	−7.6 ± 5.2 *	2.77 ± 1.1 *	0.5 ± 1.0 *	57 (37.7)
Karimi et al. [55]	Retrospective	259	19.3 ± 6.0 *	13.5 ± 3.3 *^,1^	−5.8 ± 4.8 *	2.6 ± 0.1 *	1.1 ± 1.3 *^,1^	106 (40.9) ^2^
Mansouri et al. [59]	Prospective	113	20.0 ± 7.5 *	14.1 ± 3.7 *	−6.4 ± 5.9 *	2.0 ± 1.3 *	0.6 ± 0.9 *	58 (45)
Scheres et al. [63]	Retrospective	41	19.2 ± 4.4 *	13.8 ± 3.8 *	−5.4 ± 4.1 *	2.5 ± 1.4 *	0.9 ± 1.2 *	8 (20)
Subaşı et al. [69]	Retrospective	30	20.4 ± 4.8 *	14.8 ± 1.9 *	−6.4 ± 1.2 *	3.1 ± 1.0 *	0.9 ± 1.1 *	13 (43.3)
Rauchegger et al. [71]	Retrospective	79	23.4 ± 7.9 *	14.8 ± 4.4	29(30–41) ^⁑^	2.7 ± 1.1 *	1.0 ± 1.2 *	37 (62)
Wanichwecharungruang and Ratprasatporn [84]	Retrospective	77	21.6 ± 4.0	14.6 ± 3.5 *	32.4 ^⁑,†^	2.1 ± 1.4	0.5 ± 0.7 *	10 (17.5)
Lewczuk et al. [85]	Retrospective	72	24.8 ± 8.0 *	17.5 ± 5.8 *^,3^	−7.3 ± 7.0 *	Not reported	Not reported	43 (67.2)
Nicolau et al. [89]	Retrospective	186	18.1 ± 5.1 *	12.6 ± 3.1 *	−5.5 ± 4.2 *	2.5 ± 1.1 *	1.7 ± 1.7 *	25 (13%)
Szigiato et al. [94]	Retrospective	141	23.3 ± 7.0 *	13.3 ± 4.7 *	−10.0 ± 6.0 *	3.4 ± 0.8 *	1.9 ± 1.5 *	54 (38.3)
Gillmann et al. [98] POAG PEX	Prospective	57 53	19.8 ± 5.8 * 19.8 ± 8.2 *	14.5 ± 3.6 * 14.2 ± 3.8 *	−5.3 ± 4.8 * −5.6 ± 6.4 *	1.9 ± 1.6 * 2.0 ± 1.3 *	0.6 ± 0.9 * 0.4 ± 0.7 *	42.8 43.2
Vukmirovic et al. [101]	Retrsopective	262	20.40 ± 6.31 *	Not reported	Not reported	2.70 ± 1.01 *	0.6 ± 0.9	111 (42.4)

^1^ Month-18 IOP. ^2^ Postoperative bleb needling or antimetabolite injection. ^3^ IOP at the last study visit (mean follow-up period was 26.87  ±  15.33 months). ^†^ Data about standard deviation was not provided. * Mean ± Standard deviation. ^⁑^ Percentage. Abbreviations: IOP: intraocular pressure; N: number of eyes; NOHM: number of ocular hypotensive medications; PEX: Pseudoexfoliative glaucoma.

**Table 4:** A comparison of the long-term follow-up clinical outcomes of XEN45.

Study	Type of Study	N	Length of Study (months)	Preoperative IOP (mmHg)	Final IOP	IOP Lowering (%)	Mean Reduction in Ocular Hypotensive Medication
Lenzhofer et al. [19]	Prospective	34	48	22.5 ± 4.2 *	13.4 ± 3.1 *	40.4	1.2
Gillmann et al. [60] XEN alone XEN + Phaco	Prospective Prospective	26 76	36 36	21.0 ± 7.4 * 20.0 ± 6.9 *	12.9 ± 2.9 * 12.9 ± 3.4 *	38.6 35.5	2.1 1.4
Nuzzi et al. [80]	Retrospective	23	36	24.9 ± 6.1 *	19.6 ± 2.1 *	21.3	Not reported
Reitsamer et al. [86]	Retrospective	76	36	20.7 ± 5.1 *	13.9 ± 4.3 *	32.9	1.4
Gabbay et al. [87]	Retrospective	205	36	22.6 ± 7.0 *	14.0 ± 2.9 *	38.1	2.0
Capelli et al. [93]	Retrospective	34	36	23 (19–28) **	Not reported	Not reported	Not reported
Marcos-Parra et al. [100]	Retrospective	63	36	19.1 ± 5.0 *	14.9 ± 3.8 *	−4.2 ± 4.7 *	−1.9 ± 0.8 *

Abbreviations: IOP: intraocular pressure; N: number of eyes. * Mean ± Standard deviation. ** Median (Interquartile range).

**Table 5:** A comparison of the clinical outcomes of XEN45 solo and in combination with cataract surgery (Phacoemulsification).

Study	N	Follow-Up (months)	Preop IOP, mm Hg	Final IOP, mm Hg	Final IOP Lowering	Mean Preoperative NOHM	Mean NOHM, Last Visit
Ozal et al. [37] ^1^ XEN XEN + Phaco	9 6	12	36.7 ± 4.1 * 35.2 ± 3.2 *	17.0 ± 4.2 * 15.5 ± 2.3 *	−19.7 ± 4.2 * −19.7 ± 2.8 *	3.7 ± 0.5 * Not estimable	0.3 ± 0.9 * Not estimable
Lenzhofer et al. [19] XEN XEN + Phaco	35 29	48	22.5 ± 6.5 * 23.4 ± 6.3 *	13.2 ± 5.2 * 12.7 ± 6.9 *	−9.5 ± 5.9 * −13.7 ± 6.6 *	3.0 ± 0.9 * 1.4 ± 0.6	0.8 ± 0.9 * 0.1 ± 0.4
Reitsamer et al. [45] XEN XEN + Phaco	106 79	24	21.7 ± 3.8 * 21.0 ± 3.4 *	15.4 ± 4.2 * 14.9 ± 4.5 *	−6.3 ± 4.0 * −6.1 ± 4.0 *	2.7 ± 0.9 * 2.9 ± 1.0	1.2 ± 1.2 * 1.4 ± 1.3 *
Kalina et al. [47] XEN XEN + Phaco	20 27	12	24.2 ± 8.2 * 21.0 ± 6.5 *	13.0 ± 4.5 * 13.6 ± 2.9 *	−11.2 ± 6.6 * −7.4 ± 5.0 *	Not reported	Not reported
Parra et al. [48] XEN XEN + Phaco	17 48	12	22.2 ± 6.8 * 18.0 ± 4.5 *	N.A.	−6.7 (−10.4 to −3.0) ** −3.5 (−5.0 to −2.0) **	2.5 ± 0.8 * 2.1 ± 0.9 *	0.2 ± 0.6 * 0.1 ± 0.3 *
Laborda-Guirao et al. [52] XEN XEN + Phaco	40 40	12	21.8 ± 5.3 * 20.1 ± 5.1 *	−14.4 (−15.7 to −13.2) ** −14.9 (−15.8 to −14.1) **	−6.8 ± 0.9) ^⁑^ −5.9 ± 0.9) ^⁑^	Not reported	Not reported
Karimi et al. [55] XEN XEN + Phaco	187 72	18	19.6 (18.8−20.5) ** 18.3 (17.0−19.7) **	13.5 (12.3−14.7) ** 14.0 ^†^	Not reported	Not reported	Not reported
Gillmann et al. [60] XEN XEN + Phaco	26 66	36	21.0 ± 7.4 * 20.0 ± 6.9 *	12.9 ± 2.9 * 12.9 ± 3.4 *	−8.1 ± 5.6 * −7.1 ± 5.4 *	2.4 ± 1.5 * 1.9 ± 1.2 *	0.3 ± 0.8 * 0.5 ± 0.9 *
Olgun et al. [65] XEN XEN + Phaco	51 45	24	24.4 ± 4.3 * 24.8 ± 3.5 *	14.2 ± 2.2 * 13.4 ± 1.4 *	−10.2 ± 3.5 * −11.4 ± 2.7 *	3.4 ± 0.5 * 3.4 ± 0.4 *	2.2 ± 2.0 * 1.8 ± 1.7 *
Theilig et al. [67] XEN XEN + Phaco	48 52	12	24.4 ± 6.6 * 24.8 ± 6.9 *	16.9 ± 5.9 * 16.4 ± 4.2 *	−7.5 ± 6.2 * −8.6 ± 5.3 *	3.1 ± 1.2 * 2.8 ± 1.1 *	1.5 ± 1.4 * 0.9 ± 1.4 *
Fea et al. [70] XEN XEN + Phaco	115 56	12	25.0 ^†^ 21.4 ^†^	15.8 ^†^ 15.4 ^†^	−8.8 ± 7.5 * −4.5 ± 8.4 *	3.0 ± 1.0 * 2.5 ± 1.0 *	0.5 ^†^ 0.5 ^†^
Schargus et al. [79] XEN XEN + Phaco	38 42	24	24.1 ± 4.7 * 25.4 ± 5.6 *	15.7 ± 3.0 * 14.7 ± 3.2 *	−8.4 ± 3.9 * −10.7 ± 4.6 *	3.3 ± 0.8 * 2.9 ± 0.6 *	1.2 ± 0.8 * 1.0 ± 0.4 *
Eraslam et al. [81] XEN XEN + Phaco	26 32	12	23.7 ± 6.0 * 24.4 ± 7.2 *	16.3 ± 3.0 * 16.4 ± 2.3 *	−7.4 ± 4.7 * −8.0 ± 5.3 *	2.9 ± 0.7 * 2.9 ± 0.6 *	1.0 ± 0.9 * 0.8 ± 0.8 *
Reitsamer et al. [86] XEN XEN + Phaco	98 76	36	20.4 ± 4.7 * 20.4 ± 5.5 *	14.2 ^†^ 13.6 ^†^	−6.4 ^†^ −6.7 ^†^	2.6 ± 1.0 * 2.4 ± 1.0 *	1.1 ± 1.3 * 1.2 ± 1.1 *
Marcos-Parra et al. [100] XEN XEN + Phaco	37 117	36	21.2 ± 6.2 * 18.4 ± 4.3 *	14.5 ± 4.0 * 14.9 ± 3.1 *	−3.1 ± 0.8 ^⁑^ −3.8 ± 0.4 ^⁑^	2.6 ± 0.7 * 1.9 ± 0.8 *	0.4 ± 0.8 *^,1^ 0.2 ± 0.5 *

^†^ Data about standard deviation was not provided. * Mean ± Standard deviation. ** Mean (95% confidence interval). ^⁑^ Mean ± Standard error of the mean. ^1^ Greater reduction in the XEN-alone than in the XEN + Phaco group (mean difference: 0.4 drugs; 95% CI: 0.1 to 0.7; *p* = 0.0134). Abbreviations: IOP: intraocular pressure; NOHM: number of ocular hypotensive medications.

**Table 6:** A comparison of the clinical outcomes of XEN45 and trabeculectomy. Adapted from Chen et al. [12], Wang et al. [107], and Sheybani et al. [108].

Study	XEN		Trabeculectomy		MD (95% CI) between Surgeries ^1^
	MD	SD	MD	SD	
Parra et al. [48]	−4.34	9.3	−7.73	8.97	0.37 (0.01 to 0.73)
Teus et al. [58]					
Chen et al. [12]	−7.2	9.2	−10.5	9.2	−3.30 (−6.08 to −0.52)
Wang et al. [107]	−8.8	5.2	−8.5	5.3	−0.06 (−0.86 to 0.75)
Olgun et al. [65]	−11	6.4	−16	9.7	0.63 (0.17 to 1.09)
Wagner et al. [72]	−8.5	5.3	−8.8	5.2	−0.30 (−4.51 to 3.91)
Wanichwecharungruang and Ratprasatporn [84]	−7	3.8	−10	5	−3.0 (−4.65 to −1.35)
Sheybani et al. [108]	−8.7 *	5	−10.8	4.7	−2.1 (−3.9 to −0.3)
Schlenker et al. [109]	−11	0.74	−11	7.29	0.0 (−0.21 to 0.21)
Sacchi et al. [110]	−13.38	2.76	−15.17	3.2	0.59 (0.04 to 1.15)
Olgun et al. [111]	−11	6.4	−16	9.7	−5.00 (−8.86 to −1.14)

^1^ Random effect model. * Extracted directly from the study. NP: not provided.

## Data Availability

Not applicable.
